# *Serratia* Bacteraemia in the North Denmark Region: A Population‐Based Study (2010–2024)

**DOI:** 10.1111/apm.70252

**Published:** 2026-08-24

**Authors:** Ida M. Thiele, Kirstine K. Søgaard, Jacob Bodilsen, Hans Linde Nielsen

**Affiliations:** ^1^ Department of Clinical Microbiology Aalborg University Hospital Gistrup Denmark; ^2^ Department of Clinical Medicine Aalborg University Gistrup Denmark; ^3^ Department of Infection Diseases Aalborg University Hospital Aalborg Denmark

**Keywords:** bacteraemia, epidemiology, *Serratia* infections, *Serratia liquefaciens*, *Serratia marcescens*

## Abstract

Population‐based data on *Serratia* bacteraemia are limited. We conducted a population‐based cohort study of patients with a first‐time episode of *Serratia* bacteraemia in the North Denmark Region between 1 January 2010 and 30 June 2024 (catchment population ~590,000). Clinical and microbiological data were obtained from medical records and the regional microbiological database. A total of 188 patients were identified. The median age was 70 years (IQR 60–78), 66% were male and 48% had a high Charlson Comorbidity Index score (≥ 3). The annual incidence remained stable at 2.1 per 100,000 person‐years (95% CI 1.8–2.5). Bacteraemia was community‐acquired in 38% of patients, healthcare‐associated in 2% and hospital‐acquired in 37%. The most common foci of infection were the urinary tract (25%), hepatobiliary tract (20%) and respiratory tract (14%), while a focus was not identified in 28% of patients. Thirty‐day all‐cause mortality was 24%. In an exploratory adjusted Poisson regression analysis, increasing age (RR 1.02, 95% CI 1.00–1.05) and high comorbidity (RR 2.58, 95% CI 1.01–6.63) were associated with increased mortality, whereas acquisition was not. *Serratia* bacteraemia predominantly affected older patients with substantial comorbidity and was associated with considerable mortality. Host‐related factors appeared to be important determinants of outcome.

## 1 Introduction

*Serratia* spp. are motile, facultative anaerobic, Gram‐negative bacilli belonging to the order *Enterobacterales* and the family *Yersiniaceae* [1]. Among them, *Serratia marcescens* is the most frequently implicated in human disease and is notable for producing the red pigment prodigiosin, first identified in 1819 by the pharmacist Bartolomeo Bizio [2, 3]. Historically regarded as non‐pathogenic, *S. marcescens* occurs naturally in soil and water and is frequently isolated from hospital wastewater systems, reflecting its ecological adaptability and environmental persistence [4].

The genus *Serratia* includes several species capable of causing human disease. Recent genomic analyses have demonstrated that *S. marcescens* belongs to the *S. marcescens* complex, which also includes *S. nematodiphila* , *S. bockelmannii*, *S. ureilytica* and *S. nevei* [5]. Due to the high genomic similarity among species within this complex, reliable differentiation by MALDI‐TOF MS is limited [6, 7]. Consequently, clinical isolates within the *S. marcescens* complex are often reported collectively. Other *Serratia* spp., such as *Serratia liquefaciens* , have also been associated with invasive human infections, although they occur less frequently.

Most epidemiological descriptions of *Serratia* bacteraemia originate from hospital‐based cohorts, particularly neonatal and intensive care units (ICU) or from reports of nosocomial outbreaks [8, 9]. Only a few population‐based studies have been published from Canada and Australia, and these were based on cohorts assembled more than two decades ago [10, 11]. This study examined the incidence and clinical characteristics of *Serratia* bacteraemia in the North Denmark Region from 2010 to 2024.

## 2 Material and Methods

### 2.1 Study Population and Setting

This population‐based cohort study was conducted in the North Denmark Region and included all patients with a first‐time episode of *Serratia* bacteraemia. The study period extended from 1 January 2010 to 30 June 2024, covering a catchment population of approximately 590,000 inhabitants, corresponding to about 10% of the Danish population. In Denmark, healthcare is publicly funded and freely available to all residents at the point of delivery. Each citizen is assigned a unique 10‐digit civil personal registration (CPR) number, which includes date of birth and sex and enables linkage between laboratory data, clinical records and administrative registries [12].

### 2.2 Microbiological Methods

Microbiological data were retrieved from the regional laboratory database (WWBakt, Autonik AB, Nyköping, Sweden) at the Department of Clinical Microbiology, Aalborg University Hospital, which provides microbiological services for the entire North Denmark Region [13]. The blood culture system used was the Bact/ALERT system (bioMerieux, Marcy l'Étoile, France) until 2015, after which it was replaced by the BACTEC system (Becton, Dickinson and Company, Franklin Lakes, NJ, USA). Each blood culture set consisted of three bottles: two aerobic and one anaerobic. Bacterial identification to species level was performed according to routine clinical practice using biochemical methods and/or MALDI‐TOF MS (Bruker Daltonik, Bremen, Germany). Isolates identified as *S. marcescens* were considered to represent the *S. marcescens* complex. Antimicrobial susceptibility testing (AST) was performed using the disc diffusion method and interpreted according to clinical breakpoints defined by the European Committee on Antimicrobial Susceptibility Testing (EUCAST).

### 2.3 Clinical Data and Definitions

Clinical data were obtained by review of medical records and included clinical characteristics, diagnostic examinations, antimicrobial treatment and outcomes, including all‐cause 30‐day mortality. Comorbidities were assessed using the Charlson Comorbidity Index (CCI) and categorised as low (0), moderate (1–2), or severe (≥ 3) [14]. Information on indwelling urinary or intravascular catheters, long‐term vascular access devices and joint prostheses was also recorded.

Bacteraemia was categorised as community‐acquired if blood cultures were obtained within 48 h of hospital admission; healthcare‐associated if infection occurred within 30 days of hospital contact or was associated with an indwelling device, medical procedure, instrumentation or cytotoxic‐induced neutropenia; and hospital‐acquired if blood cultures were obtained ≥ 48 h after admission [13]. Polymicrobial bacteraemia was defined as the concurrent isolation of *Serratia* spp. and one or more other clinically relevant pathogens from the same blood culture. All other clinical specimens obtained within 1 week before or after the *Serratia*‐positive blood culture were also recorded and categorised according to anatomical site.

Using all available clinical, microbiological and radiological information, the presumed focus of infection was classified as hepatobiliary, other intra‐abdominal, urinary tract, skin and soft tissue, pulmonary, other or unknown. The unknown category included cases with sparse or non‐specific clinical symptoms, or insufficient diagnostic information to reliably determine the focus of infection. Empirical antimicrobial therapy was considered adequate if it included piperacillin–tazobactam, a carbapenem, an aminoglycoside or ciprofloxacin. Definitive antimicrobial therapy was considered adequate if the administered agent was reported as susceptible (S) according to AST.

### 2.4 Statistical Analysis

Descriptive statistics were used to characterise the study population according to age, sex, comorbidity and infection focus. Continuous variables were summarised as medians with interquartile ranges (IQRs), and categorical variables as counts and percentages. There were no missing values. Annual incidence rates of first‐time *Serratia* bacteraemia were calculated per 100,000 person‐years using population data obtained from Statistics Denmark [15]. Exact Poisson 95% confidence intervals (CIs) were calculated for incidence rates. Thirty‐day all‐cause mortality was calculated from the date of the first *Serratia*‐positive blood culture. In an exploratory analysis, a modified Poisson regression model was used to estimate adjusted relative risk (RR) and 95% CIs for factors associated with 30‐day mortality, including age, sex, CCI category and acquisition category [16]. Kaplan–Meier survival curves were also constructed according to CCI category and acquisition category. Statistical analyses were performed using Stata/MP version 19 (StataCorp, College Station, TX, USA).

## 3 Results

### 3.1 Incidence and Demographics

A total of 188 patients with a first‐time episode of *Serratia* bacteraemia were identified during the study period. Six patients experienced a second episode, with a median interval of 50 days (IQR: 41–52); these recurrent episodes were not characterised further. The annual incidence of *Serratia* bacteraemia remained stable throughout the study period, with an overall incidence of 2.1 per 100,000 person‐years (95% CI 1.8–2.5). Annual incidence estimates with exact Poisson 95% confidence intervals are shown in Figure S1. The annual number of blood culture sets is shown in Figure S2. No apparent seasonal variation was observed (data not shown).

### 3.2 Baseline Characteristics

**FIGURE 1:**
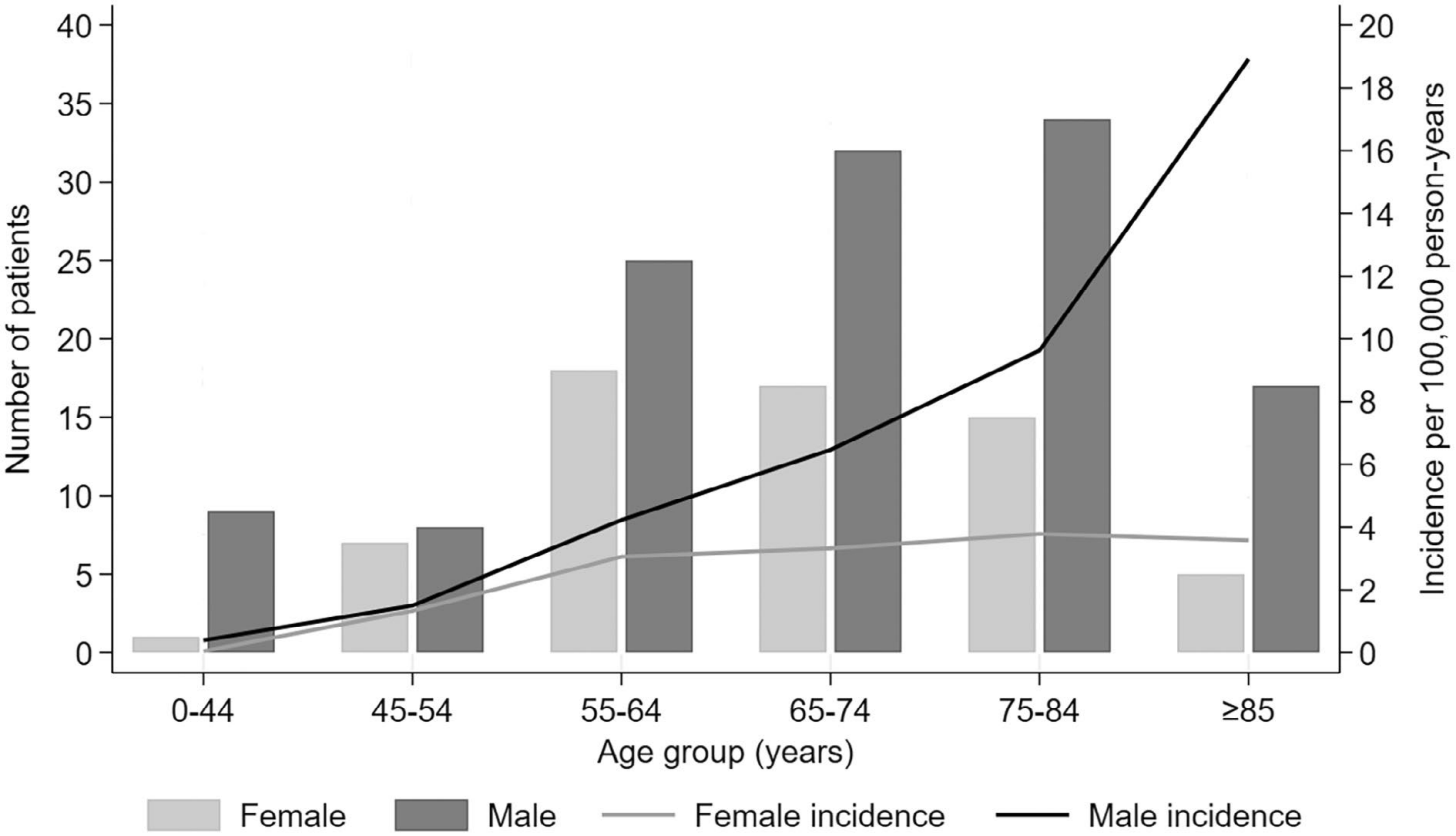
Age‐specific distribution and incidence of first‐time episode of *Serratia* bacteraemia^a^ in the North Denmark Region, 1 January 2010 to 30 June 2024. ^a^Bars represent the number of patients with a first episode of *Serratia* bacteraemia, and lines represent incidence rates per 100,000 person‐years.

The median age at first episode was 70 years (IQR: 60–78; range 0–96 years), and 125 (66%) patients were male. Three patients (all male) were younger than 1 year of age; these represented three individual cases occurring at different time points, with no evidence of a neonatal outbreak. The incidence increased markedly with age in both sexes, although the increase was more pronounced among men (Figure 1). Patients had a high burden of comorbidity, as reflected by the CCI score (Table 1). Overall, 90 (48%) patients had a high CCI score, while 65 (35%) had a moderate score and 33 (1%) had a low score. Common comorbidities included cardiovascular disease (44%) and diabetes mellitus (27%). Malignancy was present in 78 (41%) patients, predominantly solid tumours, which were almost equally divided between metastatic and non‐metastatic disease, whereas haematological malignancies were less common (9 patients, 5%). Immunosuppressive treatment was recorded in 26 (14%) patients receiving systemic corticosteroids and 16 (9%) receiving cytostatic therapy. Indwelling medical devices were present in a substantial proportion of patients, most commonly indwelling urinary catheters (49 patients, 26%), followed by pacemakers (10 patients, 5%) and prosthetic heart valves (8 patients, 4%).

**TABLE 1:** Baseline characteristics of 188 patients with a first episode of *Serratia* bacteraemia in the North Denmark Region, 1 January 2010 to 30 June 2024.

Characteristic	*n* (%) or median (IQR)
Age, years	70 (60–78)
Female sex	63 (34)
Charlson comorbidity index score	
0 (low)	33 (1)
1–2 (moderate)	65 (35)
≥ 3 (high)	90 (48)
Selected comorbidities^a^	
Cardiovascular disease	83 (44)
Diabetes mellitus	50 (27)
Chronic obstructive pulmonary disease	29 (15)
Kidney disease	21 (11)
Malignancy	78 (41)
Metastatic solid tumour	35 (19)
Localised solid tumour	34 (18)
Lymphoma	6 (3)
Leukaemia	3 (2)
Indwelling prosthetic material	
Pacemaker	10 (5)
Prosthetic heart valve	8 (4)
Joint prosthesis	5 (3)
CNS shunt	4 (2)
Endovascular graft	3 (2)
Immunosuppressive treatment	
Systemic corticosteroids	26 (14)
Cytostatic therapy	16 (9)

Abbreviation: CNS, central nervous system.

^a^ Some patients had more than one comorbidity.

### 3.3 Clinical Characteristics

Among the 188 patients, the infection was community‐acquired in 72 (38%), healthcare‐associated in 46 (2%) and hospital‐acquired in 70 (37%) (Table 2). No outbreaks were identified during the study period among neonatal or adult patients. Most patients were admitted to medical departments (131 patients, 70%), while 49 (26%) were admitted to surgical departments. A total of 46 (24%) patients were admitted to the intensive care unit (ICU), the majority of whom had hospital‐acquired bacteraemia (37 patients, 80%). Of these, 28 (61%) had their first *Serratia*‐positive blood culture obtained while in the ICU, whereas the remaining 18 (39%) were transferred to the ICU after the initial blood culture.

**TABLE 2:** Site of admission and focus of infection according to acquisition of *Serratia* bacteraemia in the North Denmark Region, 1 January 2010 to 30 June 2024.

	Overall (*n* = 188)	Community‐acquired (*n* = 72)	Healthcare‐associated (*n* = 46)	Hospital‐acquired (*n* = 70)
Site of admission, *n* (%)				
Surgical department	49 (26)	8 (11)	9 (20)	32 (46)
Medical department	131 (70)	64 (89)	37 (80)	30 (43)
Neonatal	3 (2)	—	—	—
Other	3 (2)	—		—
Intensive care unit^a^	46 (24)	7 (10)	2 (4)	37 (53)
Focus of infection^b^, *n* (%)				
No focus identified	52 (28)	23 (32)	9 (20)	20 (29)
Urinary tract	47 (25)	28 (39)	13 (28)	6 (9)
Hepatobiliary	38 (20)	9 (13)	13 (28)	16 (23)
Pulmonary	27 (14)	5 (7)	4 (9)	18 (26)
Intravascular catheter‐related	17 (9)	2 (3)	5 (11)	10 (14)
Other intra‐abdominal	6 (3)	1 (1)	3 (7)	2 (3)
Other^c^	9 (5)	4 (6)	1 (2)	4 (6)
Targeted antibiotic treatment^d^, *n* (%)				
Piperacillin‐tazobactam	99 (53)	34 (47)	30 (65)	35 (50)
Meropenem	18 (10)	1 (1)	1 (2)	16 (23)
Ciprofloxacin	100 (53)	47 (65)	27 (59)	26 (37)
No targeted treatment	17 (9)	8 (11)	4 (9)	5 (7)

^a^ Patients admitted to the intensive care unit may also be included in surgical or medical departments.

^b^ Ten patients involved more than one focus of infection.

^c^ Includes skin and soft tissue infections (*n* = 3), bone and joint infections (*n* = 2), ventriculitis (*n* = 2), and intravenous drug use–associated infections (*n* = 2).

^d^ Missing treatment data (*n* = 6). Ciprofloxacin and piperacillin–tazobactam were used either as combination therapy or as sequential therapy with intravenous piperacillin–tazobactam followed by oral ciprofloxacin.

The focus of the infection was not identified in 52 (28%) patients, with similar proportions across acquisition category (Table 2). Among patients with an identifiable source, urinary tract infections (UTIs) were the most common, comprising 48 (25%) episodes. These were predominantly community‐acquired, accounting for 28 (39%) of community‐acquired episodes, whereas UTIs were uncommon in hospital‐acquired cases (6 episodes, 9%). Hepatobiliary infections accounted for 38 (20%) episodes and were more frequently observed in healthcare‐associated (13 episodes, 28%) and hospital‐acquired cases (16 episodes, 23%) than in community‐acquired cases (9 episodes, 13%). Pulmonary infections were identified in 27 (14%) patients and occurred predominantly in hospital‐acquired bacteraemia, representing 18 (26%) of these episodes. Intravascular catheter‐related infections were identified in 17 (9%) patients, the majority of which were hospital‐acquired (10 episodes, 14%). Other intra‐abdominal infections were uncommon, accounting for 6 (3%) episodes. Nine patients (5%) were classified as having other foci of infection, whereas 10 patients had more than one focus of infection.

When stratified by sex, UTIs were more frequent among men (36/125, 29%) than women (11/63, 17%). Hepatobiliary infections occurred equally in absolute numbers in men and women (19 episodes each), but were proportionally more common among women (30% vs. 15%). The prevalence of pulmonary infections was 16% among men and 11% among women. The proportions of patients with unknown focus of infection were similar among men and women (28% vs. 27%).

### 3.4 Microbiological Findings

Among the 188 patients with a first‐time episode of *Serratia* bacteraemia, a total of 201 *Serratia*‐positive blood culture sets were identified. In total, 206 *Serratia* isolates were recovered from these blood culture sets, including 194 belonging to the *S. marcescens* complex, eleven *S. liquefaciens* and one *S. fonticola* . Seven blood culture sets yielded more than one isolate; six contained two isolates of the *S. marcescens* complex with differing antimicrobial susceptibility profiles, while one yielded both *S. liquefaciens* and *S. fonticola* .

Polymicrobial bacteraemia was identified in 29 (15%) patients and was evenly distributed across acquisition categories. The most frequent focus among these patients was hepatobiliary infection, accounting for approximately half of polymicrobial episodes. The most commonly co‐isolated bacteria were *Klebsiella* spp. (*n* = 8), streptococci (*n* = 6) and *Escherichia coli* (*n* = 3).

In 97 (52%) patients, *Serratia* spp. were also identified from one or more additional clinical specimens. The most frequent sources were urine (*n* = 46) and respiratory samples (*n* = 28), followed by intravascular catheter samples (*n* = 20) and intra‐abdominal or hepatobiliary drainage (*n* = 10). Less frequent sources included skin swabs (*n* = 6), pleural drainage (*n* = 5), and tissue or abscess samples (*n* = 4). Susceptibility was consistent with an inducible AmpC phenotype, while susceptibility to the remaining clinically relevant antibiotics was high. Specifically, 96% of isolates were susceptible to ciprofloxacin and piperacillin–tazobactam, 97% to gentamicin and 100% to meropenem.

### 3.5 Antimicrobial Treatment

Empirical antimicrobial therapy active against *Serratia* spp. had been initiated in 117 (62%) patients at the time of blood culture collection. Among the remaining 71 (38%) patients, 54 had not yet received antimicrobial therapy, whereas 17 had received empirical therapy without activity against *Serratia* spp. Piperacillin–tazobactam and ciprofloxacin were the most commonly used targeted antimicrobial agents, administered in 99 (53%) and 100 (53%) patients, respectively. Meropenem was used in 18 (10%) patients. Seventeen patients (9%) did not receive targeted antimicrobial therapy for *Serratia* bacteraemia, and treatment data were unavailable for six (3%) patients.

### 3.6 Mortality

Among the 188 patients with a first episode of *Serratia* bacteraemia, 46 (24%) died within 30 days of the first *Serratia*‐positive blood culture, and 22 (12%) died within 7 days. In the exploratory modified Poisson regression analysis adjusted for age, sex, CCI, and acquisition category, increasing age (RR 1.02, 95% CI 1.00–1.05) and high comorbidity (RR 2.58, 95% CI 1.01–6.63) were associated with increased 30‐day mortality, whereas acquisition category was not. Kaplan–Meier survival curves similarly demonstrated lower survival among patients with high comorbidity, whereas survival appeared comparable across acquisition categories (Figures S3 and S4). Among patients who died within 30 days, the focus of infection was not identified in 20 (43%) patients, while eight (17%) patients had a urinary tract focus, seven (15%) a hepatobiliary focus and six (13%) a pulmonary focus. The remaining five patients (12%) had other infection foci.

## 4 Discussion

In this population‐based study, *Serratia* bacteraemia was an uncommon but clinically significant infection, with an annual incidence of 2.1 per 100,000 person‐years and a 30‐day all‐cause mortality of 24%. The infection occurred predominantly among older adults with substantial comorbidity, including a high prevalence of cardiovascular disease, diabetes and malignancy. Urinary tract and hepatobiliary infections were the most common identified foci, although more than one‐quarter of episodes had no identifiable source. Notably, mortality was associated with increasing age and comorbidity burden rather than acquisition category, highlighting the importance of host‐related factors in determining outcome.

The observed incidence aligns with previous reports from Canada and Australia, as well as national laboratory surveillance data from England, Wales and Northern Ireland, which have reported rates in the range of 1–3 per 100,000 person‐years [10, 11, 17]. Similar to previous studies of *Serratia* and other Gram‐negative bacteraemia, two‐thirds of patients in our cohort were male [10, 11, 18, 19]. Our study further demonstrates that these patients had a substantial burden of comorbidity, with nearly half having a high CCI score. Cardiovascular disease, diabetes and malignancy were particularly common. These findings support the view of *Serratia* spp. as opportunistic pathogens that primarily affect vulnerable patients with underlying chronic disease and frequent healthcare exposure. The higher incidence among older adults likely reflects the accumulation of comorbidities, repeated healthcare contacts, and greater use of invasive procedures among older adults [11, 17, 19]. However, the stable incidence of *Serratia* bacteraemia contrasts with the increasing incidence reported for bacteraemia overall and for common urinary tract‐associated pathogens, such as *E. coli* , in Denmark [20]. This may indicate that the epidemiology of *Serratia* bacteraemia differs from that of more common Gram‐negative bloodstream infections, potentially reflecting differences in reservoirs, clinical risk factors and transmission pathways.

Among identifiable foci, the urinary tract was the most common source of infection, particularly among community‐acquired episodes. UTIs were more common among men, whereas hepatobiliary infections were proportionally more common among women, although these observations should be interpreted cautiously given the limited sample size. The hepatobiliary tract was the second most frequent focus, accounting for one‐fifth of episodes.

Previous studies have generally reported lower proportions of hepatobiliary infections in *Serratia* bacteraemia [21, 22], although differences in study populations and case definitions may contribute to this variation. A notable finding was the high proportion of episodes without an identified focus of infection. Similar observations have been reported in studies of *Serratia* bacteraemia and Gram‐negative bloodstream infections more broadly [18, 19]. The absence of a defined focus may reflect non‐specific clinical presentation or rapidly progressive disease. Although unknown focus was more common among patients who died within 30 days, this likely reflects disease severity, as the focus of infection may not have been established before death. Pulmonary infections accounted for a smaller proportion of episodes but occurred predominantly among hospital‐acquired cases. This aligns with previous studies identifying *Serratia* spp. as causes of nosocomial respiratory tract infections, particularly among critically ill patients and those requiring prolonged hospitalisation or intensive care [3, 19, 23].

In our cohort, the majority of episodes were caused by species belonging to the *S. marcescens* complex, consistent with earlier reports indicating that *S. marcescens* is the dominant pathogenic species within the genus [5, 10, 17, 24]. A smaller proportion of cases were attributed to *S. liquefaciens* , and these episodes showed similar clinical and microbiological characteristics. Polymicrobial bacteraemia was observed in 15% of episodes, most commonly in association with hepatobiliary infections, likely reflecting translocation from the gastrointestinal tract. This is lower than the 45.7% reported by Deniz et al. in hospital‐acquired *Serratia* bacteraemia [21].

Nearly all isolates were susceptible to piperacillin‐tazobactam, ciprofloxacin and gentamicin, and all were susceptible to meropenem. This is consistent with studies from Australia and Canada, which generally report low levels of acquired resistance in non‐outbreak *Serratia* spp. [10, 11]. In contrast, lower susceptibility to piperacillin‐tazobactam and ciprofloxacin has been reported in other settings [21, 22, 25].

All‐cause mortality was 12% within 7 days and 24% within 30 days. This is comparable to previously reported mortality rates of 12%–25% for Gram‐negative bacteraemia [17, 24, 26, 27], and 10%–30% for *Serratia* bacteraemia [21, 22]. In the exploratory adjusted analysis, increasing age and high comorbidity were associated with increased 30‐day mortality, whereas acquisition category was not. Together, these findings suggest that host‐related factors may be more important determinants of outcome than the setting in which the infection was acquired.

The strengths of this study include its population‐based design, long study period, and comprehensive inclusion of community‐acquired, healthcare‐associated and hospital‐acquired *Serratia* bacteraemia. The use of a unified microbiological database and detailed medical record review allowed for comprehensive characterisation of both microbiological and clinical features. However, several limitations should be acknowledged. First, the retrospective design relied on historical medical records and may have resulted in incomplete clinical information and potential misclassification of infection focus. Second, although exploratory adjusted analyses of mortality were performed, the relatively limited number of deaths restricted the number of variables that could be included in multivariable models, and the results should therefore be interpreted cautiously. Third, residual confounding, particularly by disease severity, cannot be excluded. Finally, the inability to reliably distinguish species within the *S. marcescens* complex may have obscured species‐specific differences.

In conclusion, this study provides updated population‐based data on *Serratia* bacteraemia. The infection occurred predominantly among older patients with substantial comorbidity and was associated with considerable 30‐day mortality. Urinary tract and hepatobiliary infections were the most common identified foci. The absence of a clear difference in mortality across acquisition categories, together with the association between mortality, age and comorbidity burden, supports the importance of host‐related factors in determining outcome. These findings emphasise the need for early recognition, thorough diagnostic evaluation and appropriate empirical antimicrobial therapy in this patient population.

## Funding

The authors have nothing to report.

## Ethics Statement

The study was approved by the Regional Council of the North Denmark Region (approval no. 1‐45‐72‐4220‐24) and registered in the regional research registry (ID: F2024‐109) in compliance with Danish data protection regulations.

## Consent

The authors have nothing to report.

## Conflicts of Interest

I.M.T. has received speaker honoraria from AstraZeneca (2022) and conference support from Tillots Pharma (2025), unrelated to the present study.

## Supporting information


**Figure S1:** Annual incidence of first‐time *Serratia* bacteraemia per 100,000 person‐years in the North Denmark Region, 2010–2024*. Error bars represent exact Poisson 95% confidence intervals.
**Figure S2:** Annual number of blood culture sets received at the Department of Clinical Microbiology, Aalborg University Hospital, 2010–2024*.
**Figure S3:** Kaplan–Meier estimates of 30‐day survival following first‐time *Serratia* bacteraemia according to Charlson Comorbidity Index (CCI) category. Time was measured from the date of the first *Serratia*‐positive blood culture. CCI categories were defined as low (score 0), moderate (score 1–2) and high (score ≥ 3).
**Figure S4:** Kaplan–Meier estimates of 30‐day survival following first‐time *Serratia* bacteraemia according to acquisition category (community‐acquired, healthcare‐associated and hospital‐acquired). Time was measured from the date of the first *Serratia*‐positive blood culture.

## Data Availability

The data are not publicly available due to Danish data protection legislation but may be available from the corresponding author on reasonable request and with relevant institutional approvals.
