# Supplementary material for: Serratia Bacteraemia in the North Denmark Region: A Population‐Based Study (2010–2024)
Source: APMIS. 2026 Aug 24;134(8):e70252. doi: 10.1111/apm.70252 (PMC13501340; doi:10.1111/apm.70252)
Supplement: Supplementary file 1 [file APM-134-0-s001.docx]

**Supplementary material**

**Supplementary Figure 1.** Annual incidence of first-time *Serratia* bacteraemia per 100,000 person-years in the North Denmark Region, 2010–2024*. Error bars represent exact Poisson 95% confidence intervals.

*to 30 June 2024.

**Supplementary Figure 2.** Annual number of blood culture sets received at the Department of Clinical Microbiology, Aalborg University Hospital, 2010–2024*.

*to 30 June 2024.

**Supplementary Figure 3.** Kaplan–Meier estimates of 30-day survival following first-time *Serratia* bacteraemia according to Charlson Comorbidity Index (CCI) category. Time was measured from the date of the first *Serratia*-positive blood culture. CCI categories were defined as low (score 0), moderate (score 1–2), and high (score ≥3).

**Supplementary Figure 4.** Kaplan–Meier estimates of 30-day survival following first-time *Serratia* bacteraemia according to acquisition category (community-acquired, healthcare-associated, and hospital-acquired). Time was measured from the date of the first *Serratia*-positive blood culture.
